# A comparative study of post-operative endophthalmitis between immediate versus delayed management of eventful cataract extraction

**DOI:** 10.12669/pjms.36.4.1911

**Published:** 2020

**Authors:** Hina Loya, Hunain Razzak Ghoghari, Syed Fawad Rizvi

**Affiliations:** 1 Dr. Hina Loya, MBBS. Layton Rahamatullah Benevolent Trust (LRBT), Free Base Eye Hospital, Korangi 2 ½, Karachi, Pakistan; 2 Dr. Hunain Ghoghari, MBBS, MRCSEd (Ophth). Layton Rahamatullah Benevolent Trust (LRBT), Free Base Eye Hospital, Korangi 2 ½, Karachi, Pakistan; 3 Prof. Syed Fawad Rizvi, MCPS (Ophth), FCPS (Ophth). Layton Rahamatullah Benevolent Trust (LRBT), Free Base Eye Hospital, Korangi 2 ½, Karachi, Pakistan

**Keywords:** Postoperative Endophthalmitis (POE), Posterior Capsular Rupture (PCR), Phacoemulsification, Secondary Intraocular Lens, Extracapsular Cataract Extraction

## Abstract

**Objective:**

To compare the incidence of postoperative endophthalmitis after eventful cataract surgery i.e. posterior capsular rupture, in patients managed with anterior vitrectomy and intra ocular lens implantation intraoperatively to patients managed with anterior vitrectomy and intraocular lens implantation two weeks after the posterior capsule rupture.

**Methods:**

This comparative study was conducted at Layton Rahamatullah Benevolent Trust, Free Base Eye Hospital Korangi, Karachi from February 2017 to December 2018. The study included two groups, group A had patients with intra ocular lens implanted after posterior capsule rupture intraoperatively, while Group B had patients with intra ocular lens implanted after two weeks of initial surgery. Incidence rate of post-operative endophthalmitis was compared between two groups, which happened within six weeks after intra ocular lens implantation surgery.

**Results:**

Total number of cataract surgeries that were performed during the study was 37,969. Incidence of postoperative endophthalmitis was 0.0019%. The study enrolled patients with complicated cataract surgeries that were 3508 (0.09%). Out of which incidence of post-operative endophthalmitis that occurred in group A was 0.007% and group B was 0.002%. (p value <0.05). The study also found that complicated extracapsular cataract extraction with intraocular lens implantation had more cases of endophthalmitis compared to phacoemulsification with intraocular lens.

**Conclusion:**

This study showed that management of posterior capsular rupture through anterior vitrectomy and secondary intraocular lens implantation after two weeks has less chances of postoperative endophthalmitis.

## INTRODUCTION

Cataract extractions are the most commonly performed intraocular surgery in the field of ophthalmology.^1^ Use of newer machines have made cataract extraction easier to perform.^2^ There are many types of cataract surgery, in which phacoemulsification is most commonly performed. Other types include Extracapsular Cataract Extraction (E.C.C.E), Intracapsular Cataract Extraction (I.C.C.E), and Small Incision Cataract Surgery (S.I.C.S). The most common intraoperative complication of cataract surgery is Posterior Capsule Rupture (PCR).^3^ PCR is the breach in continuity of posterior surface of human lens capsule. This can occur in removing the lens, during Intraocular Lens (IOL) implant, and cortex aspiration. PCR can be managed intraoperatively or later on after two to three weeks with secondary IOL implant, under cover of topical and systemic antibiotic and anti-inflammatory.

The most serious and distressful complication after any intraocular surgery is endophthalmitis. In about 90% of cases endophthalmitis occurs after extraction of cataract as it is performed most frequently.^4^ Its prevalence is from 0.012% to 0.2%.^5^ Acute Post-Operative Endophthalmitis (POE) is more common than chronic. Acute POE occurs within six weeks of cataract surgery, while chronic POE occurs within six weeks to nine months. Diagnosis of POE is entirely clinical. Blurred vision, pain, swollen eyelids, hypopyon, red eye, and media haze are common clinical features of POE. Vitritis is the hallmark of the disease.^6^,^7^ Despite proper sterilization, preoperative antibiotic, specialized technique and improvement in surgical environment, endophthalmitis still occurs.^8^ Diabetes mellitus, surgical complications i.e. PCR and vitreous loss are the primary risk factor for this complication. Other factors include age, site of incision, improper sterilization, and ocular surface diseases.^9^

All previous studies have reported the incidence of POE, but did not work on the comparison between immediate and delayed management POE following PCR due to cataract surgery. The study was conducted to compare the incidence rate of POE in patients managed immediately intraoperatively following PCR during cataract surgery to patients managed with anterior vitrectomy and IOL implantation two weeks after the PCR due to cataract surgery.

## METHODS

This comparative study was conducted at Layton Rahmatullah Benevolent Trust eye hospital Korangi from February 2017 to December 2018. Prior ethical approval was obtained by the research ethical committee of the institute. This prospective study included 55 patients with acute POE who were diagnosed within six weeks of eventful cataract surgery. Informed consent was obtained from all the subjects.

POE was diagnosed clinically based on anterior segment activity, diminished vision, and ocular pain. All patients with acute POE after PCR were included in the study. In both the groups all anti-septic measures were taken to prevent POE includimg 5% povidone-iodine solution placed in conjunctival fornix prior to surgery. In addition, preparation of the skin around the eye with 10% povidone-iodine solution was done and injection cefuroxime was added in the irrigating fluid. Patients were divided into two groups. Group A included cases who underwent eventful phacoemulsification and E.C.C.E, and were managed intraoperatively with anterior vitrectomy and with Kelman anterior chamber IOL. Group B encompassed cases who came with POE and were managed after two weeks of initial surgery with Kelman anterior chamber IOL.

Patients with chronic endophthalmitis, traumatic endophthalmitis and endogenous endophthalmitis were excluded from the study.

### Statistical Analysis

The statistical analysis of the data was done by the software Statistical Package for Social Sciences (SPSS) version 21. Mean and the standard deviation were calculated for quantitative variables like age of patient. Frequency and percentage charts were designed for type of cataract extraction, and incidence of POE after complicated cataract surgery. A non para-metric test was run to compare the incidence rate of POE between the groups. A p-value ≤ 0.05 was considered statistically significant.

## RESULTS

**Fig.1:**
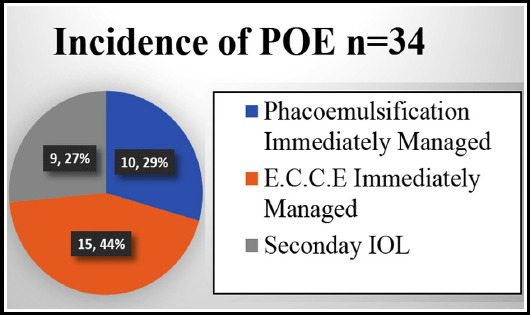
Incidence of POE in Different Type of Cataract Surgeries.

Total number of cataract surgery that was done during the duration of study was 37969. Incidence of PCR was 3,508 (0.09%). Within 24 months, 75 cases were reported with endophthalmitis. Out of which 55 had acute POE. Thirty-four cases were enrolled in the study as they had PCR and presented within six weeks of cataract surgery. Mean age of patient was 60 ± 5.4 years. Out of 34 cases, 21 were males and 13 were females. Male (51.8%) were affected more than females (46.4%). The below pie chart shows the incidence of endophthalmitis as follows. (Fig.1)

**Fig.2:**
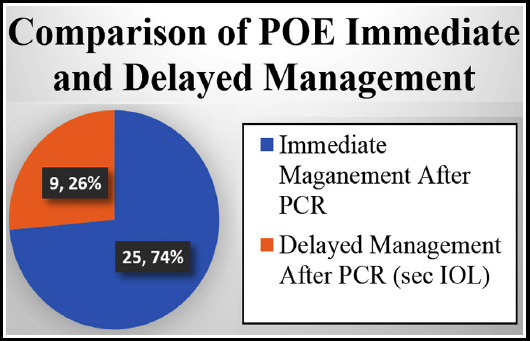
Comparison of POE between Two Groups.

Comparing the two groups on basis of incidence of endophthalmitis, group A presented with more cases than group B which was statistically significant (p value <0.05) as shown in Fig.2.

## DISCUSSION

In our study we compared two groups with acute POE who underwent PCR during cataract surgery. On comparison, the incidence of POE was more in patients who were immediately managed intraoperatively (group A) than patients who were managed two weeks after PCR (group B) i.e. 25 and nine cases respectively.

In our study total incidence of POE after cataract surgery was 0.0019% and mean age of patient was 60 ± 5.4 years. Male (51.8%) were affected more than females (46.4%). On the contrary, 52 (0.11%) patients reported POE after cataract surgery in a three-year study done by Yanan Zhu et al in China. Mean age of these patients was 68.3 years old (range 28–92) and females were affected more than males.^10^

As mentioned above incidence of endophthalmitis in our study was 0.0019%. Incidence of POE after eventful cataract surgery was 0.09%. On the contrary, the incidence of POE in developed countries ranges from 0.012 to 0.076%^11^-^17^ which is more than in our study.

Incidence of endophthalmitis varied according to the type of intraocular surgery i.e. 0.04-0.07%, and 0.02% after secondary IOL implantation. MS Nazimul Hussein et al in 2001 reported incidence of endophthalmitis as 0.07-0.12% while 0.4% after secondary IOL which was more than our results.^18^ In another 14-year review by Pei Chang et al, 56 cases had postoperative endophthalmitis, out of which this condition developed in 46 cases after cataract surgery while only one patient had endophthalmitis after secondary IOL implant.^19^

Our study also found that in complicated cataract surgeries, E.C.C.E had more chance of endophthalmitis than phacoemulsification and secondary IOL i.e. 13, 8, and 9 cases respectively. The authors could not find any studies in published literature comparing the rates of endophthalmitis in eventful cataract surgeries based on the type of cataract surgery performed, similar to how it has been calculated in the current study; therefore, comparison was made with studies which looked at the significance of cataract surgery types in their overall endophthalmitis cases. F. Koc et al in 2002, reported that E.C.C.E had more chance of endophthalmitis than phacoemulsification and scleral fixated IOL i.e. 18, 8 and 2 respectively. 20 Whereas in two study, one conducted by L spadea et al showed that POE is more common after secondary IOL than phacoemulsification with IOL implant (0.1-0.3%),^21^ and in 5-year study at Bascom Palmer eye institute showed incidence of POE is less with E.C.C.E. (0.072%) than secondary IOL. (0.3%). One probable reason for the variation in the results suggested by the study authors is the higher number of cases undergoing E.C.C.E.^22^ It has been observed that in our study the less incidence of POE in group B can be due to pre-operative use of systemic and topical anti-inflammatory and antibiotics.

### Limitations of the study

The duration of study is less. Large scale and multicenter study should be done.

## CONCLUSION

This study showed that delayed management of PCR with anterior vitrectomy and secondary IOLs had less chance of acute POE. It can be due to a more comprehensive approach to an already complicated surgery along with the meticulous use of systemic and topical antibiotic and anti-inflammatory that renders the surgical field more sterile and it gives enough time for the anterior vitreous face to settle and allowing better clearance of vitreous from the wound hence removing the source of infection and better wound closure.

### Author’s Contribution

**HL** conceived, designed, did data collection and manuscript writing.

**HG** did statistical analysis & editing of manuscript.

**SFR** did the review and takes the responsibility and is accountable for all aspects of the work in ensuring that questions related to the accuracy or integrity of any part of the work are appropriately investigated and resolved.
